# Food malabsorption/intolerance complaints triggered by primary epiploic appendagitis

**DOI:** 10.17179/excli2019-1667

**Published:** 2019-08-29

**Authors:** Wolfgang J. Schnedl, Pia Reittner, Dietmar Enko, Harald Mangge

**Affiliations:** 1 General Internal Medicine Practice, Dr.-Theodor-Körner-Strasse 19b, A-8600 Bruck, Austria; 2 Diagnostikum Sued-West, Weblinger Guertel 25, A-8054 Graz, Austria; 3 Institute of Laboratory Medicine, General Hospital Steyr, Sierninger Straße 170, A-4400 Steyr, Austria; 4 Clinical Institute of Medical and Chemical Laboratory Diagnosis, Medical University of Graz, Auenbruggerplatz 30, A-8036 Graz, Austria

**Keywords:** primary epiploic appendagitis, computed tomography, fructose malabsorption, histamine intolerance

## Abstract

Primary epiploic appendagitis (PEA) is an uncommon and self-limiting cause of acute or subacute abdominal complaints. The diagnosis of PEA, with its characteristic appearance, is made with computed tomography (CT). This report describes a patient seven months after a CT-confirmed diagnosis of PEA. Because of persistent and recurring, functional, non-specific abdominal complaints, food intolerance/malabsorption was investigated. Fructose malabsorption combined with histamine intolerance was found. A registered dietician helped develop an individually-tailored diet to address the problem. Within four days of beginning the fructose-free and histamine-reduced diet, the patient's complaints resolved. In conclusion, abdominal symptoms caused by fructose malabsorption and histamine intolerance may have been triggered by PEA in this patient.

## Abbreviations

PEA, primary epiploic appendagitis; CT, computed tomography; H_2, _hydrogen; DAO, diamine oxidase; HIT, histamine intolerance

## Introduction

Primary epiploic appendagitis (PEA) is a rare, benign, self-limiting inflammation of epiploic appendages, which are adipose structures protruding from the colon. The use of cross-sectional imaging computed tomography (CT) for the primary evaluation of abdominal pain has increased the recognition of PEA over the past years. The pain in PEA is reported as being dull, constant and non-migrating (Schnedl et al., 2011[9]). Adverse reactions to ingested food are a result of the intolerance/malabsorption of carbohydrates (lactose and fructose), proteins (gluten), and biogenic amines (histamine). In food intolerance/malabsorption, one or more food ingredients cannot be digested and/or absorbed properly within the gastrointestinal tract (Enko et al., 2016[2]). Symptoms include persistent and recurring, functional, non-specific abdominal complaints, such as: bloating, semisolid stools, intermittent diarrhea and migrating abdominal pain. This report describes a patient who seven months earlier had been diagnosed with PEA using CT. Due to abdominal complaints, we investigated food intolerance/malabsorption. Fructose malabsorption combined with histamine intolerance was diagnosed. An individually-tailored fructose-free and histamine-reduced diet resolved the patient's symptoms.

## Case Report

**Figure 1:**
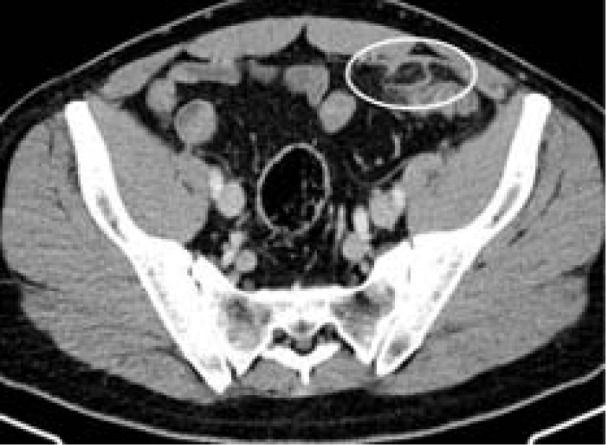
Contrast enhanced axial abdominal CT demonstrating primary epiploic appendagitis adjacent to the sigmoid colon 7 months prior to presentation

A 48-year-old white male patient presented with persistent and recurring, functional, non-specific abdominal complaints, including bloating, semisolid stools and intermittent diarrhea up to a maximum of three times per day. Physical examination revealed a bloated abdomen and migrating, non-localized pain and tenderness. Abdominal sonography showed gas distention, but no other abnormalities. Anamnesis revealed that he had had a CT-diagnosed epiploic appendagitis seven months prior (Figure 1(Fig. 1)). The patient was concerned about the continuing PEA because he experienced persistent and recurring abdominal symptoms after the initial diagnosis. Through anamnesis the symptoms were correlated with the ingestion of food of drinks. Consequently, food malabsorption/intolerance was suspected.

We used hydrogen (H_2_) breath tests (Gastrolyzer, Bedfont Scientific Inc., Kent, England) for the evaluation of lactose intolerance and fructose malabsorption. During a hydrogen breath test with a drink containing 25 g of fructose, the exhalation demonstrated increasing H_2_ values of up to 86 parts per million (normal < 20) and the diagnosis of fructose malabsorption was confirmed. Diamine oxidase (DAO) in the serum was measured with the radio extraction assay DAO Rea 100 (Sciotec Diagnostic Technologies, Tulln, Austria) and was determined to be 3 U/mL (normal > 10). This DAO value, combined with abdominal complaints and the correlation with the ingestion of histamine-containing food, indicated histamine intolerance. The lactose breath test with a 50 g lactose load showed no increase in exhaled H_2_ and simultaneously measured blood glucose increased by > 20 mg/dl. An enzyme-linked IgA immunosorbent assay (ELISA, Serion, Würzburg, Germany) showed the absence of *Helicobacter pylori *infection. In the screening for celiac disease, antibodies against tissue transglutaminase with anti-tTG IgA ELISA (Euro Diagnostica AB, Malmö, Sweden) were not found. A CT of the abdomen performed as follow up to the PEA diagnosed 7 months prior demonstrated no anomalies. Triglycerides were 228 mg/dl (normal < 150), but all of the other routine laboratory parameters, including erythrocyte sedimentation rate and liver and pancreas enzymes, were within normal limits. A colonoscopy also showed no abnormalities.

**Figure 2:**
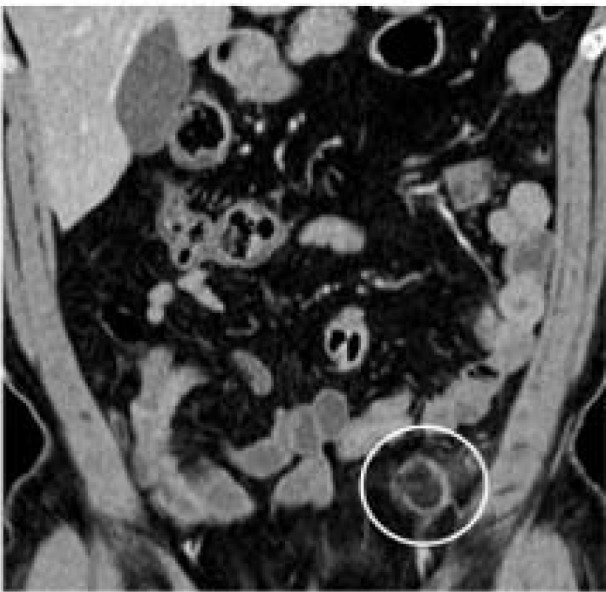
Longitudinal abdominal CT with contrast enhancement demonstrating primary epiploic appendagitis adjacent to the sigmoid colon 7 months prior to presentation

Seven months earlier, an abdominal CT with intravenous contrast medium demonstrated an oval lesion with a maximum diameter of 1.7 x 0.7 cm located at the sigmoid colon. The diagnosis of PEA was arrived at based on the hyperattenuated rim, surrounded by fat stranding, which indicated an inflamed and thickened visceral peritoneum surrounding the fat-containing appendage (Figure 2(Fig. 2)).

With the diagnosis of combined food intolerance/malabsorption, the patient received written information on fructose malabsorption and histamine intolerance. A registered dietician helped develop an individually-tailored diet to address the symptomatology and to ensure nutritional adequacy. Within 4 days of beginning the fructose-free and histamine-reduced diet, the patient's complaints resolved. Written informed consent was obtained for all procedures, which were in accordance with the Declaration of Helsinki and the recommendations of the local ethics committee.

## Discussion

Appendices epiploicae are 50 to 100 subserosal fat pouches lining the entire length of the colon. They are attached to the colon wall with a vascular stalk and appear in two rows along the tenias. There is only one row along the transverse colon. This explains why cases of PEA are predominantly reported at the sigmoid and ascending colon. Clinically, PEA is accompanied by localized pain mainly in the lower left or right abdominal quadrant. This pain is described as dull, constant and non-migrating. Physical examination may show a well-localized tenderness (Schnedl et al., 2011[9]). However, the pain in PEA can be similarly identified as diverticulitis, cholecystitis or appendicitis (Singh et al., 2005[12]) because clinicians are frequently unfamiliar with PEA (Almeida et al., 2009[1]). It usually resolves within a few days to a few weeks without medical or surgical treatment.

CT is the diagnostic modality of choice for identifying PEA with its characteristic and diagnostic appearance (Rioux and Langis, 1994[8]). The use of CT scans for the primary evaluation of acute and subacute abdominal pain is increasing, but, so far, only about 200 publications on PEA exist. PEA is assumed to be a localized sterile inflammation in and adjacent to a distorted epiploic appendage. This torsion may lead to ischemia, infarction, aseptic fat necrosis, and spontaneous venous thrombosis (Ozdemir et al., 2010[6]). Regular non-tortured epiploic appendages cannot be seen on CT scans. A secondary epiploic appendagitis can develop along with the inflammation of neighboring organs, such as cholecystitis, pancreatitis, diverticulitis, or appendicitis (Lorente et al., 2017[4]). 

Usually bowel habits are not disturbed by PEA, although a few reports described postprandial fullness, bloating, constipation, diarrhea and vomiting (Schnedl et al., 2014[10]). In this patient we documented the difference between the symptoms from PEA and food intolerance/malabsorption. Seven months of a bloated abdomen, semisolid stools and the migrating, non-localized pain were found to be caused by fructose malabsorption and histamine intolerance.

In fructose malabsorption, this monosaccharide is not adequately absorbed by the epithelial glucose transporters, mainly GLUT-5, in the intestinal mucosa. Fructose reaches the large intestine where it serves as a bacterial substrate. This results in fermentation with hydrogen production (Gibson et al., 2007[3]). Within the gastrointestinal tract, the enzyme diamine oxidase (DAO) appears to be central to the degradation of ingested histamine. Abdominal complaints in histamine intolerance (HIT) result mainly from the consumption of histamine-containing food and the reduced ability of DAO to digest histamine. Serum DAO values may be used for the diagnosis of HIT (Mušič et al., 2013[5]), but there is no established correlation with gastrointestinal DAO activity (Reese et al., 2017[7]). Various combinations of intolerance/malabsorption, as in this patient, have been proposed to cause persistent and recurring, functional, non-specific abdominal symptoms (Enko et al., 2016[2]).

## Conclusion

Numerous mechanisms - visceral hypersensitivity, irregular gut motility, abnormal brain-gut interactions, and infectious agents - are thought to be related to the initiation and development of functional, non-specific abdominal symptoms (Shariati et al., 2019[11]). In conclusion, abdominal complaints caused by concomitant fructose malabsorption and histamine intolerance may have been triggered by PEA in this patient. 

## Conflict of interest and funding

Wolfgang J. Schnedl received speaking honoraria from Sciotec. All of the authors declare no conflicts of interest. The authors have received no funding for this manuscript.
